# Supplementary material for: Resolving the full spectrum of human genome variation using Linked-Reads
Source: Genome Res. 2019 Apr;29(4):635–45. doi: 10.1101/gr.234443.118 (PMC6442396; doi:10.1101/gr.234443.118)
Supplement: Supplemental Material [file supp_gr.234443.118_Supplemental_longranger-2.2.zip › longranger-2.2.tar.bz2!/lr2.2-public/lib/stan/cmdstan/stan/doc/api/html/precomp__v__vari_8hpp_source.html]

Stan: src/stan/agrad/rev/internal/precomp\_v\_vari.hpp Source File


|  |  |
| --- | --- |
|  | Stan  2.5.0  probability, sampling & optimization |


- Main Page
- Related Pages
- Namespaces
- Classes
- Files

- File List
- File Members

All Classes Namespaces Files Functions Variables Typedefs Enumerations Enumerator Friends Macros Pages

- src
- stan
- agrad
- rev
- internal

precomp\_v\_vari.hpp

Go to the documentation of this file.

1 #ifndef STAN\_\_AGRAD\_\_REV\_\_INTERNAL\_\_PRECOMP\_V\_VARI\_HPP

2 #define STAN\_\_AGRAD\_\_REV\_\_INTERNAL\_\_PRECOMP\_V\_VARI\_HPP

3

4 #include <stan/agrad/rev/vari.hpp>

5 #include <stan/agrad/rev/internal/v\_vari.hpp>

6

7 namespace stan {

8  namespace agrad {

9

10  // use for single precomputed partials

11  class precomp\_v\_vari : public op\_v\_vari {

12  protected:

13  double da\_;

14  public:

15  precomp\_v\_vari(double val, vari\* avi, double da)

16  : op\_v\_vari(val,avi),

17  da\_(da) {

18  }

19  void chain() {

20  avi\_->adj\_ += adj\_ \* da\_;

21  }

22  };

23

24  }

25 }

26 #endif

stan::agrad::precomp\_v\_vari::da\_

double da\_

**Definition:** precomp\_v\_vari.hpp:13

stan::agrad::op\_v\_vari::avi\_

vari \* avi\_

**Definition:** v\_vari.hpp:11

vari.hpp

stan::agrad::precomp\_v\_vari::precomp\_v\_vari

precomp\_v\_vari(double val, vari \*avi, double da)

**Definition:** precomp\_v\_vari.hpp:15

stan::agrad::op\_v\_vari

**Definition:** v\_vari.hpp:9

v\_vari.hpp

stan::agrad::vari

The variable implementation base class.

**Definition:** vari.hpp:28

stan::agrad::precomp\_v\_vari

**Definition:** precomp\_v\_vari.hpp:11

stan::agrad::precomp\_v\_vari::chain

void chain()

Apply the chain rule to this variable based on the variables on which it depends. ...

**Definition:** precomp\_v\_vari.hpp:19

stan::agrad::vari::adj\_

double adj\_

The adjoint of this variable, which is the partial derivative of this variable with respect to the ro...

**Definition:** vari.hpp:43


---

[ Stan Home Page ]

*© 2011–2014,
Stan Development Team.*
